# Perioperative Intravenous Acetaminophen Attenuates Lipid Peroxidation in Adults Undergoing Cardiopulmonary Bypass: A Randomized Clinical Trial

**DOI:** 10.1371/journal.pone.0117625

**Published:** 2015-02-23

**Authors:** Frederic T. Billings IV, Michael R. Petracek, L. Jackson Roberts II, Mias Pretorius

**Affiliations:** 1 Division of Clinical Pharmacology, Department of Medicine, Vanderbilt University Medical School, Nashville, Tennessee, United States of America; 2 Department of Cardiac Surgery, Vanderbilt University Medical School, Nashville, Tennessee, United States of America; 3 Department of Pharmacology, Vanderbilt University Medical School, Nashville, Tennessee, United States of America; 4 Department of Anesthesiology, Vanderbilt University Medical School, Nashville, Tennessee, United States of America; San Raffaele Scientific Institute, ITALY

## Abstract

**Background:**

Cardiopulmonary bypass (CPB) lyses erythrocytes and induces lipid peroxidation, indicated by increasing plasma concentrations of free hemoglobin, F_2_-isoprostanes, and isofurans. Acetaminophen attenuates hemeprotein-mediated lipid peroxidation, reduces plasma and urine concentrations of F_2_-isoprostanes, and preserves kidney function in an animal model of rhabdomyolysis. Acetaminophen also attenuates plasma concentrations of isofurans in children undergoing CPB. The effect of acetaminophen on lipid peroxidation in adults has not been studied. This was a pilot study designed to test the hypothesis that acetaminophen attenuates lipid peroxidation in adults undergoing CPB and to generate data for a clinical trial aimed to reduce acute kidney injury following cardiac surgery.

**Methods and Results:**

In a prospective double-blind placebo-controlled clinical trial, sixty adult patients were randomized to receive intravenous acetaminophen or placebo starting prior to initiation of CPB and for every 6 hours for 4 doses. Acetaminophen concentrations measured 30 min into CPB and post-CPB were 11.9±0.6 μg/mL (78.9±3.9 μM) and 8.7±0.3 μg/mL (57.6±2.0 μM), respectively. Plasma free hemoglobin increased more than 15-fold during CPB, and haptoglobin decreased 73%, indicating hemolysis. Plasma and urinary markers of lipid peroxidation also increased during CPB but returned to baseline by the first postoperative day. Acetaminophen reduced plasma isofuran concentrations over the duration of the study (P = 0.05), and the intraoperative plasma isofuran concentrations that corresponded to peak hemolysis were attenuated in those subjects randomized to acetaminophen (P = 0.03). Perioperative acetaminophen did not affect plasma concentrations of F_2_-isoprostanes or urinary markers of lipid peroxidation.

**Conclusions:**

Intravenous acetaminophen attenuates the increase in intraoperative plasma isofuran concentrations that occurs during CPB, while urinary markers were unaffected.

**Trial Registration:**

ClinicalTrials.gov NCT01366976

## Introduction

Cardiopulmonary bypass (CPB) lyses erythrocytes, increasing plasma concentrations of free hemoglobin.[1–4] Free hemoglobin undergoes hemeprotein redox cycling that, in the absence of antioxidants, initiates oxidation of free and phospholipid-esterified fatty acids.[5] Markers of lipid peroxidation are increased in patients with hemoglobinemia during cardiac surgery and in those that develop postoperative acute kidney injury (AKI).[1,6] Thus, strategies aimed at attenuating hemeprotein-associated lipid peroxidation could potentially reduce postoperative organ dysfunction, such as AKI following CPB. In this regard, acetaminophen inhibits hemoglobin-induced oxidation of arachidonic acid.[5] *In vitro*, the half maximal inhibitory concentration (IC_50_) for acetaminophen reduction of arachidonic acid oxidation is 17.7±2.5μM, well within the therapeutic range (10–30 μg/mL plasma; 67–200μM) in humans.[5] In an animal model of rhabdomyolysis-induced kidney injury, acetaminophen significantly decreased markers of lipid peroxidation and preserved kidney function.[5] Reduced hemeprotein redox cycling of myoglobin accounted for these benefits. Furthermore, in pediatric patients undergoing CPB, acetaminophen attenuated the increase in plasma isofuran concentrations but did not affect urinary makers of lipid peroxidation, serum concentrations of creatinine, or urine concentrations of neutrophil gelatinase-associated lipocalin (NGAL).[4] The effect of acetaminophen on oxidative damage in adults undergoing CPB is unknown and deserves further study since intraoperative oxidative damage independently predicts postoperative AKI[7] and 500,000 patients undergo cardiac surgery each year. This study tested the hypothesis that acetaminophen reduces oxidant injury in adults undergoing elective cardiac surgery requiring CPB.

## Methods

The protocol for this trial and supporting CONSORT checklist are available as supporting information; see S1 CONSORT Checklist and S1 Protocol. Sixty adult elective cardiac surgery patients were recruited (ClinicalTrials.gov Identifier: NCT01366976). This parallel designed study was approved by the Vanderbilt University Institutional Review Board for Research on Human Patients and conducted according to the Declaration of Helsinki. All subjects provided written informed consent. Patients were enrolled by the research nurse at the time of the preoperative evaluation for surgery. The study period was from January 2012 until April 2013. Patients were eligible for the study if he/she was 18–80 years of age and undergoing elective cardiac surgery requiring CPB. Patients were excluded for the following reasons: 1) allergy to acetaminophen, 2) evidence of severe hepatic impairment (history of liver cirrhosis or total bilirubin >2.0mg/dL) 3) evidence of impaired renal function (serum creatinine >2.0mg/dL) or 4) pregnancy. This study adhered to the CONSORT reporting guidelines.[8]

### Protocol

Recruited subjects were randomly assigned by the investigational pharmacy to acetaminophen or matching placebo treatment in a 1:1 ratio. Zhiguo Zhao, Department of Biostatistics, generated the block randomization schedule for the 2 treatment arms with four patients in each block of randomization. Intravenous acetaminophen (Cadence Pharmaceuticals, San Diego, CA) was administered at the FDA approved dose of 1g every 6 hours for a weight >50kg (maximum of 4g per 24 hours) or 15mg/kg every 6 hours for a weight <50kg (maximum of 75 mg/kg per 24 hours). Study drug or placebo treatment was administered every 6 hours, with the first dose in the operating room and before the onset of CPB and the second, third, and fourth doses over the following 24 hours. To ensure blinding from subjects, physicians, and study personnel, the study drug was prepared, labeled, and delivered to clinical staff by the investigational pharmacy in an identical fashion, and no study personnel were informed of treatment assignment during subject study. Study personnel were unblinded to treatment assignment after completion of the statistical analyses. No open label acetaminophen was permitted while subjects received study drug. Acetaminophen plasma concentrations were measured 30 min following initiation of CPB and post-CPB to assess acetaminophen pharmacokinetics during CPB and to allow comparisons of acetaminophen concentrations with simultaneously collected measurements of free hemoglobin and markers of lipid peroxidation. No changes were made to the methods after trial commencement, and the trial completed as planned.

### Anesthesia and Cardiopulmonary Bypass

Anesthesia management and CPB were conducted according to institutional protocols as previously described.[9] Briefly, subjects received general endotracheal anesthesia. Induction of anesthesia was achieved with either etomidate or propofol and maintained with isoflurane, fentanyl, air and oxygen. Muscle relaxation was achieved and maintained with rocuronium or vecuronium. CPB was achieved with a non-pulsatile roller pump (Medtronic, Minneapolis, MN) and a trillium-coated circuit (Medtronic, Minneapolis, MN). Heparin was used for anticoagulation during CPB at an initial dose of 300 U/kg, supplemented with additional heparin to achieve and maintain an activated clotting time (ACT) greater than 400 seconds. Heparin was neutralized with protamine sulfate after separation from CPB, at an initial dose of 250 mg, with an additional 50 mg if the ACT remained greater than 140 seconds. Subjects were transfused according to the following guidelines: packed red blood cells (PRBC) were transfused for a hematocrit less than 20% during CPB and for a hematocrit less than 27% after CPB. Platelets were transfused for ongoing microvascular bleeding despite a normalized ACT and a platelet count <100X10^9^mL^-1^. Fresh frozen plasma (FFP) was transfused for continued bleeding and an INR (international normalized ratio) greater than 1.5. Cryoprecipitate was transfused in 10 pack units if fibrinogen concentrations were less than 200 mg/dL.

### Primary Outcome

The primary outcome of the study was the oxidative stress response as measured by plasma and urine F_2_-isoprostanes and isofurans.

### Secondary Outcomes

Blood loss (chest tube drainage), number of blood products transfused, need for surgical re-exploration, mechanical ventilation hours, cardiac enzymes, aspartate aminotransferase (AST), urinary NGAL, prevalence of postoperative atrial fibrillation and acute kidney injury (AKI) and hospital length of stay were recorded. AKI was defined by Acute Kidney Injury Network (AKIN) consensus criteria AKI staging: stage 1, 0.3 mg/dL or 50% increase in serum creatinine concentration within 72 hours of surgery; stage 2, 100% increase in serum creatinine within 72 hours; and stage 3, 200% increase in serum creatinine or need for renal replacement therapy. Any patient that developed any stage of AKI within 72 hours of surgery was labeled as having postoperative AKI. [10,11]

### Blood Sampling and Biochemical Assays

Blood samples were obtained to measure free hemoglobin, haptoglobin, F_2_-isoprostanes, and isofurans. F_2_-isoprostanes and isofurans are sensitive and specific markers of lipid peroxidation *in vivo*,[5,12,13] and are increased after CPB.[6,14] Measurement of both isofuran and F_2_-isoprostane concentrations provides the most reliable approach to assess oxidative stress status under conditions of varying concentrations of oxygen, because the formation of F_2_-isoprostanes relative to isofurans is suppressed by elevated concentrations of oxygen.[13] Cardiac surgery patients are routinely administered oxygen concentrations in excess of those required to saturate hemoglobin, and this practice could increase isofuran production during surgery. All blood samples were collected on ice and centrifuged immediately at 0°C for 20 minutes. Plasma was then separated and stored at -80°C until the time of assay. Urine samples were obtained for measurement of F_2_-isoprostanes, isofurans and neutrophil gelatinase-associated lipocalin (NGAL), a marker of acute kidney injury. Blood and urine samples were collected at six time points: 1) after induction of anesthesia but prior to CPB and administration of study drug (baseline), 2) following 30 min of CPB, 3) following 60 min of CPB, 4) after protamine administration (post-bypass), 5) upon arrival in the ICU, and 6) 24 hours after administration of first acetaminophen dose on postoperative day 1 (POD1). Free hemoglobin was determined using the 2-wavelength method s previously described.[15] Haptoglobin concentrations were measured using a commercially available ELISA kit (Abcam, Cambridge, MA) according to the manufacturer’s instructions. Free F_2_-isoprostane (non-esterified) and isofuran concentrations were determined by gas chromatography-mass spectrometry (GCMS) as previously described.[13,16] Urine NGAL was measured using a commercially available ELISA assay (Bioporta Diagnostics, Gentofte, Denmark). Acetaminophen concentrations were quantified with the Roche COBAS INTEGRA 800 System analyzer (Roche Diagnostics Corporation, Indianapolis, IN).

### Statistical Analysis

Categorical data were compared between groups using Chi-squared or Fischer’s exact tests, as appropriate. Continuous baseline data were compared using Student’s *t*-test or Mann-Whitney *U* test, as appropriate. Normality of data was assessed by generating normal QQ-plots and the Kolmogorov-Smirnov test. Sample size calculations were based on preliminary plasma isofuran data from humans (55 pg/ml isofuran concentrations post CPB)[6] and the effect of acetaminophen on hemeprotein-mediated lipid peroxidation in animals (~40% reduction in markers of lipid peroxidation).[5] We hypothesized that acetaminophen would reduce peak isofuran concentrations by 22 pg/mL (40% reduction from peak concentrations). If the true difference in the acetaminophen and placebo group means is 22 pg/mL (SD = 30 pg/mL), we would need to study 30 experimental subjects and 30 control subjects to be able to reject the null hypothesis that the population means of the acetaminophen and placebo groups are equal with probability (power) 0.8. The Type I error probability associated with this test of this null hypothesis is 0.05. Repeated measures of free hemoglobin, haptoglobin and markers of lipid peroxidation were analyzed using mixed effects models with fixed effects of study drug (Acetaminophen versus Placebo) and time. We included a random subject effect and a first-order autoregressive process [AR(1)] to adjust for any errors in the mixed effects model. Because diabetes affects lipid peroxidation we included subject diabetes status as a covariate in the mixed effects model that assesses the effect of acetaminophen on lipid peroxidation.[17] A Bonferroni correction was done to adjust for multiple comparisons. A 2-tailed P value less than 0.05 was considered statistically significant. Continuous data are presented as means±SEM unless otherwise indicated. Statistical analyses were performed with the statistical package SPSS for Windows (Version 21.0, IBM, New York, NY).

## Results

### Patient Characteristics and Effects of Acetaminophen on Intraoperative and Postoperative Events

Sixty-seven patients were consented to participate in the study. Two of these subjects were excluded for failing exclusion criteria, 1 withdrew prior to randomization, 1 surgery was canceled, 1 surgery date was changed, and 2 subjects did not proceed with the study because of refusal to stop acetaminophen prior to surgery. Sixty subjects were randomly assigned to acetaminophen or placebo study drug and were included in the intention-to-treat analysis. There were no study drug administration protocol violations. Acetaminophen and placebo study groups had comparable demographic data, medical history, preoperative medications and laboratory values (Table 1). Study groups also had comparable intraoperative characteristics including type of surgery, time on CPB, cross-clamp time, use of cardioplegia, hemoconcentration use on CPB, administration of steroids in CPB pump prime, and cell saver volume administered (Table 2). Subjects in the placebo group, however, were significantly more likely to receive intraoperative red cell or platelet transfusions. AST concentrations increased in both the acetaminophen (from a median baseline of 23 to 56 U/L on POD1, P<0.001) and placebo group (from a median baseline of 24 to 54 U/L on POD1, P<0.001). Postoperative outcomes are presented in Table 3. Chest tube output, surgical re-exploration, mechanical ventilation hours, CK-MB/CK ratio, AST concentrations, postoperative atrial fibrillation, or incidence of AKI were not significantly different between subjects randomized to acetaminophen or placebo. Subjects in the acetaminophen group tended to have a shorter hospital length of stay. No in-hospital mortality occurred. Acetaminophen concentrations measured at 30min of bypass and post-bypass were 11.9±0.6 μg/mL (78.9±3.9 μM) and 8.7±0.3 μg/mL (57.6±2.0 μM), respectively.

**Table 1:** Preoperative Patient Characteristics.

Characteristic	Acetaminophen (N = 30)	Placebo (N = 30)	P-value
Age, years	64.5 (49.0, 68.4)	61.0 (54, 67.8)	0.55^a^
Gender, women N (%)	10 (33.3)	14 (46.7)	0.29^d^
Race, Caucasian N (%)	28 (93.3)	29 (96.7)	1.00^c^
Body Mass Index (kg/m^2^)	29.1±0.9	29.4±1.3	0.82^b^
Systolic blood pressure (mmHg)	130.0±3.6	134.1±3.0	0.39^b^
Diastolic blood pressure (mmHg)	74±2.3	74.4±2.4	0.92^b^
Medical History, N (%)			
Prior cardiac surgery	6 (20.0)	6 (20.0)	1.00^d^
Hypertension	18 (60.0)	17 (56.7)	0.79^d^
Atrial Fibrillation	6 (20.0)	4 (13.3)	0.49^d^
Diabetes	6 (20.7)	4 (13.3)	0.51^c^
Smoking history	10 (33.3)	12 (40.0)	0.59^d^
PVD	4 (13.3)	1 (3.3)	0.35^c^
Preoperative medications, N (%)			
β-Blockers	18 (60.0)	14 (46.7)	0.30^d^
ACE inhibitors	9 (30.0)	6 (20.0)	0.37^d^
ARB	4 (13.3)	4 (13.3)	1.00^c^
Statins	18 (60.0)	13 (43.3)	0.20^d^
Diuretic	9 (30.0)	10 (33.3)	0.78^d^
Aspirin	20 (66.7)	18 (60.0)	0.59^d^
Clopidogrel	2 (6.7)	2 (6.7)	1.00^c^
Preoperative			
Hematocrit, %	42 (40, 44)	42 (39, 43)	0.45^a^
Platelet count, (10^9^mL^-1^)	215 (192, 254)	216 (199, 280)	0.27^a^
AST, (U/L)	23 (19, 27)	24 (20, 27)	0.63^a^
Creatinine, mg/dL	0.98±0.04	0.94±0.03	0.51^b^
CKD EPI eGFR, (mL/min/1.73m^2^)	80.2±3.4	79.5±3.0	0.87^b^
Blood glucose, mg/dL	98 (89, 123)	95 (90, 104)	0.34^a^
Ejection fraction, %	60 (55, 65)	60 (55, 65)	0.80^a^

Continuous data are reported as mean± SEM or median (25^th^, 75^th^ percentile). PVD, peripheral vascular disease; ACE, angiotensin-converting enzyme; ARB, angiotensin receptor blocker; AST, aspartate aminotransferase; CKD EPI eGFR, chronic kidney disease epidemiology collaboration estimated glomerular filtration rate.[27]

^a^ Mann Whitney U test;

^b^ t-test;

^c^ Fischer Exact test,

^d^ Pearson Chi-Square test

**Table 2:** Intraoperative Patient Characteristics.

Characteristic	Acetaminophen (N = 30)	Placebo (N = 30)	P-value
Surgery Type			0.65^c^
Valvular surgery only, N (%)	24 (80.0)	24 (80.0)	
CABG surgery only, N (%)	1 (3.3)	0	
CABG and valvular surgery, N (%)	3 (10.0)	4 (13.3)	
Congenital surgery, N (%)	2 (6.7)	1 (3.3)	
Other surgery, N (%)	0	1 (3.3)	
Cardiopulmonary bypass time, min	122 (100, 148)	114 (93, 144)	0.55^a^
Cardioplegia, N (%)	13 (43.3)	15 (50.0)	0.61^c^
Cross-clamp time, min	78 (73, 99)	69 (60, 135)	0.50^a^
Hemoconcentration used, N (%)	17 (56.7)	19 (63.3)	0.60^c^
Steroids in pump prime, N (%)	20 (66.7)	25 (83.3)	0.14^c^
Transfusion in OR, N (%)			
Packed red blood cells	3 (10.0)	11 (36.7)	0.02^c^
Plasma	5 (16.7)	9 (30.0)	0.22^c^
Platelets	5 (16.7)	14 (46.7)	0.01^c^
Cryoprecipitate	3 (10.0)	3 (10.0)	1.00^b^
Cell saver blood, mL	550 (500, 760)	500 (375, 650)	0.19^a^

Continuous data are reported as median (25^th^, 75^th^ percentile).CABG, coronary artery bypass graft; OR, operating room.

^a^ Mann Whitney U test;

^b^ Fischer Exact test

^c^ Pearson Chi-Square test

**Table 3:** Postoperative Outcomes.

Characteristic	Acetaminophen (N = 30)	Placebo (N = 30)	P-value
Chest tube output in 24hrs, mL	340 (267, 461)	323 (187, 485)	0.54^a^
Re-exploration for bleeding, N (%)	1 (3.3)	1 (3.3)	1.00^c^
Mechanical ventilation, hours	5.0 (4.0, 7.5)	5.3 (3.9, 8.6)	0.68^a^
CK-MB/CK ratio POD1	6.0 (4.1, 8.2)	5.3 (3.5, 7.2)	0.24^a^
AST POD1, U/L	56 (43, 106)	54 (45, 76)	0.98^a^
AST fold increase from baseline	2.5 (2.0, 3.3)	2.4 (2.0, 2.7)	0.42^a^
Postoperative atrial fibrillation, N (%)	4 (13.3)	4 (13.3)	1.00^c^
POD1 creatinine, mg/dL	0.89±0.04	0.90±0.04	0.82^b^
POD2 creatinine, mg/dL	0.96±0.04	0.96±0.04	0.94^b^
POD3 creatinine, mg/dL	0.93±0.05	0.92±0.04	0.85^b^
Post-bypass urine NGAL, ng/mL	16.4 (11.2, 68.2)	18.9 (9.7, 175.8)	0.83^a^
Acute kidney injury†, N (%)	3 (10.0)	3 (10.0)	1.00^c^
Length of hospital stay, days	5.0 (4.0, 6.0)	5.0 (5.0, 6.0)	0.07^a^

Continuous data are reported as mean± SEM or median (25^th^, 75^th^ percentile).CK, creatine kinase; AST, aspartate aminotransferase; POD, postoperative day; NGAL, neutrophil gelatinase-associated lipocalin; †Acute kidney injury was defined as an increase in subject serum creatinine concentration of 50% or 0.3 mg/dL within 72 hours of surgery.

^a^ Mann Whitney U test;

^b^ t-test;

^c^ Fischer Exact test

### Hemolysis during CPB

**Fig 1:**
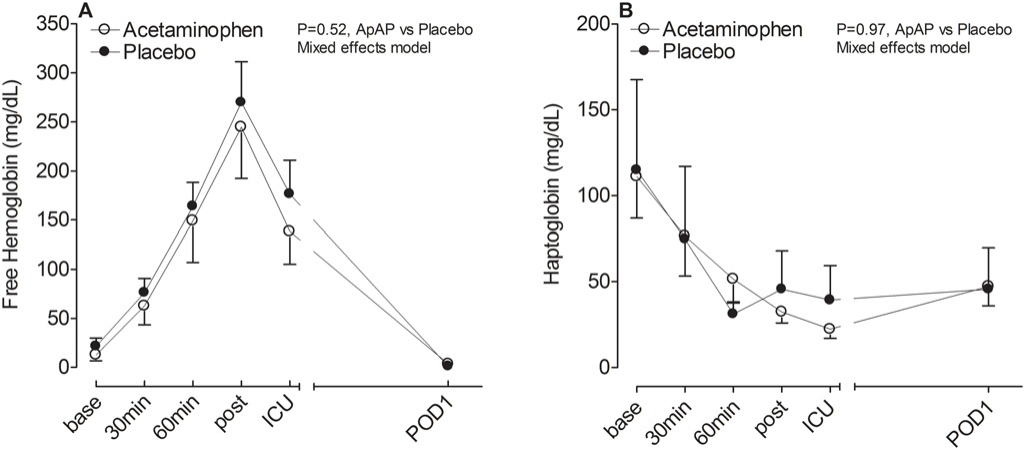
Free hemoglobin (A) and haptoglobin (B) concentrations. CPB was associated with an increase in free hemoglobin concentrations and a decrease in haptoglobin concentrations with no significant difference between the acetaminophen and placebo group. base, baseline; 30min, 30min of CPB; 60min, 60min of CPB; post, post-bypass; ICU, intensive care unit; and POD1, postoperative day 1.

CPB increased plasma free hemoglobin concentrations 15-fold (from a baseline of 17.3±5.1 to a peak of 257.3±33.1 mg/dL post-CPB, P<0.001 for effect of time on free Hb). Plasma free hemoglobin concentrations returned to baseline concentrations by POD1. Subjects that received intraoperative PRBC transfusions (N = 14) had significantly higher free hemoglobin concentrations post-bypass compared to those that did not receive intraoperative PRBCs (389.1±52.9 versus 217.2±20.4 mg/dL, P = 0.002). Even though subjects in the placebo group received more PRBC transfusions, the degree of hemolysis, quantified by measuring free hemoglobin concentrations in plasma, was not higher in the placebo group compared to the acetaminophen group (P = 0.52 for effect of study drug on free hemoglobin concentrations, Fig. 1A). As expected, there was a significant correlation between the duration of CPB and peak free hemoglobin concentrations post-bypass (correlation coefficient 0.46, P<0.001). Haptoglobin, the free hemoglobin scavenger, decreased by 73% during surgery (from baseline concentrations of 113.0±28.8 to a nadir of 30.8±10.4 mg/dL at ICU admission, P<0.001 for effect of time on haptoglobin concentrations) and remained low on POD1. There was no significant difference in the haptoglobin concentrations between the acetaminophen and placebo groups (P = 0.97 for effect of study drug, Fig. 1B).

### Lipid Peroxidation

**Fig 2:**
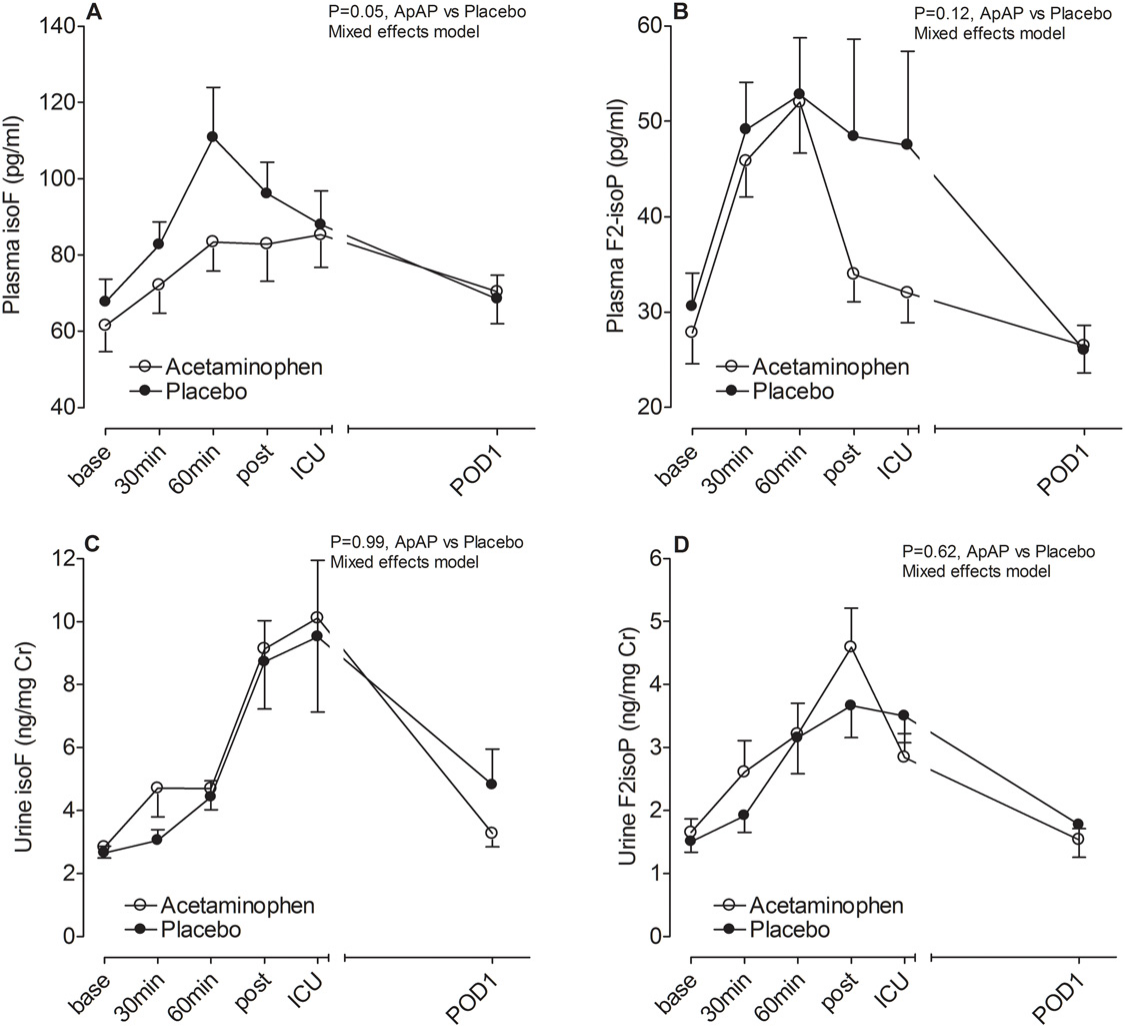
Effect of study drug on plasma and urine markers of lipid peroxidation.; isoF, isofurans; F_2_isoP, F_2_-isoprostanes; base, baseline; 30min, 30min of CPB; 60min, 60min of CPB; post, post-bypass; ICU, intensive care unit; and POD1, postoperative day 1.

Plasma isofuran and F_2_-isoprostane concentrations increased during CPB, peaked at 60min, and returned to baseline concentrations by POD1 (P<0.001 for effect of time on plasma isofuran and F_2_-isoprostane concentrations). Similar to plasma markers of lipid peroxidation, urine isofurans and F_2_-isoprostanes increased during surgery (P<0.001 for effect of time on urine isofuran and F_2_-isoprostane concentrations) but peaked later and returned to baseline by POD1. The magnitude of urine isofuran increase was greater than the magnitude of urine F_2_-isoprostane increase (4.4±1.2 vs. 2.1±0.2 fold at ICU admission, P<0.001). Perioperative intravenous acetaminophen attenuated the increase in plasma isofuran concentrations compared to placebo (Fig. 2A, P = 0.05 for effect of study drug on plasma concentrations of isofurans). This effect was most pronounced during surgery, coinciding with peak hemolysis (P = 0.03 for effect of study drug analyzing intraoperative time points only). Controlling for surgery type (CABG vs. valve surgery), in a separate mixed effects model, did not change the independent effect of acetaminophen on intraoperative plasma isofuran concentrations. Acetaminophen did not significantly affect plasma concentrations of F_2_-isoprostanes (Fig. 2B) or urinary markers of lipid peroxidation (Fig. 2C and 2D).

### Clinical Outcomes

**Fig 3:**
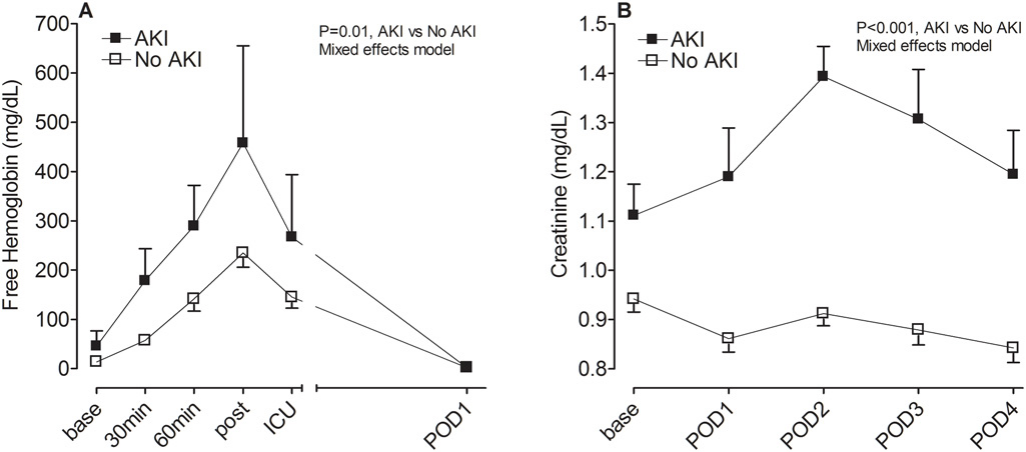
Plasma free hemoglobin (A) and creatinine (B) concentrations in subjects that developed acute kidney injury (AKI) compared to subjects that did not. base, baseline; 30min, 30min of CPB; 60min, 60min of CPB; post, post-bypass; ICU, intensive care unit; and POD, postoperative day.

Because hemolysis and lipid peroxidation have been associated with postoperative AKI in prior studies we explored the effect of acetaminophen on AKI in our study population and measured the associations between markers of oxidative damage and AKI. Six subjects (10%) developed postoperative Acute Kidney Injury Network grade I AKI. Perioperative intravenous acetaminophen did not impact postoperative creatinine concentrations, urine NGAL concentrations, or the incidence of AKI following surgery (Table 3). Subjects that developed AKI had significantly higher intraoperative free hemoglobin concentrations (Fig. 3A), peak plasma isofuran concentrations (124.5±12.8 vs. 83.7±6.1 pg/mL, P = 0.015), and serum creatinine (Fig. 3B) compared to subjects that did not develop AKI. Baseline, but not intraoperative or POD1 haptoglobin concentrations, tended to be lower in subjects that subsequently developed AKI compared to concentrations in subjects that did not develop AKI (38.1±11.8 vs. 121.3±31.8 mg/dL, P = 0.07).

## Discussion

This prospective randomized double-blind placebo-controlled clinical trial revealed that perioperative intravenous acetaminophen reduces plasma concentrations of isofurans, a robust marker of oxidative damage associated with elevated concentrations of oxygen tension, but does not appear to affect plasma concentrations of F_2_-isoprostanes or markers or lipid peroxidation in the urine during cardiac surgery. We demonstrated that CPB induces hemolysis, increasing concentrations of plasma free hemoglobin and reducing circulating concentrations of haptoglobin. These findings have important implications to the production and elimination of oxygen free radicals during cardiac surgery.

Free hemoglobin undergoes auto-oxidation to ferric hemoglobin which is then oxidized to ferryl hemoglobin, releasing a lipid radical.[18] Ferryl hemoglobin is reduced back to ferric hemoglobin and this redox cycling continues, each time generating a lipid radical, until the free hemoglobin is eliminated, often by binding to haptoglobin, forming the substrate for heme-oxygenase-1 mediated degradation.[19] Haptoglobin thus acts as an important physiological antioxidant in the kidney and reduces oxidative damage in renal tissues during hemolysis.[20] Haptoglobin concentrations decreased during CPB and remained low on the first postoperative day confirming significant intravascular hemolysis. This decrease in haptoglobin concentrations is consistent with prior studies in adults but differs from the response observed in pediatric patients undergoing CPB.[1,4,21] In our prior pediatric study we did not observe a significant decrease in intraoperative haptoglobin levels whereas in another study haptoglobin levels were only decreased 6 hours post-bypass.[1,4] One potential explanation for the difference between children and adults may be the more frequent use of plasma for CPB pump priming in pediatric patients, exogenously increasing circulating haptoglobin.

We and others have previously demonstrated that cardiac surgery and CPB increase markers of lipid peroxidation in the plasma and urine of both adult and pediatric patients during and following surgery.[4,6,14,22] In the current study we confirmed this oxidative stress response to CPB and discovered that acetaminophen attenuates the increase in intraoperative plasma isofurans but not F_2_-isoprostanes or urinary makers of lipid peroxidation. Interestingly, we made the same discovery in pediatric patients randomized to IV acetaminophen or placebo and exposed to cardiac surgery with CPB.[4] Isofurans and F_2_-isoprostanes are both markers of free radical-induced arachidonic acid peroxidation, but a relatively high oxygen tension increases the production of isofurans relative to F_2_-isoprostanes, whereas a relatively low oxygen tension increases the relative production of F_2_-isoprostanes.[13] Cardiac surgery patients are administered high concentrations of inspired oxygen, and hyperoxemia during surgery may increase the production of isofurans relative to F_2_-isoprostanes. This may provide an opportunity for acetaminophen to reduce isofuran production to a greater extent than F_2_-isoprostanes. The modest effect of acetaminophen on plasma isofurans and lack of effect on other markers of lipid peroxidation in the current study may also be explained by additional reasons. Acetaminophen was dosed intermittently, and we may not have achieved consistently high plasma concentrations during increased reactive oxygen species production. Haptoglobin levels, even though dramatically reduced during CPB, were not completely depleted and could have attenuated hemeprotein-mediated lipid peroxidation by scavenging free hemoglobin.[19,23] And last, isofurans and F_2_-isoprostanes indicate systemic oxidative damage and are not exclusive to hemeprotein redox cycling-induced lipid peroxidation, the therapeutic target of acetaminophen. Because of this, acetaminophen may not completely diminish lipid peroxidation in these patients, since other mechanisms of intraoperative oxidative damage, such as reperfusion of ischemic tissues, NADPH oxidase activation, or mitochondrial dysfunction may increase reactive oxygen species production during cardiac surgery.

Since circulating hemeproteins are known to induce kidney injury, intraoperative lipid peroxidation independently predicts postoperative AKI,[7] and acetaminophen inhibited hemeprotein-mediated lipid peroxidation and preserved kidney function in an animal model of rhabdomyolysis, we performed an exploratory analysis of postoperative AKI.[5] Acetaminophen did not affect postoperative creatinine concentrations, postoperative urinary NGAL concentrations, or the incidence of AKI. This exploratory analysis was limited by the low incidence of postoperative AKI in the current study, so we were underpowered to detect small differences in creatinine or NGAL concentrations. A significant effect of IV acetaminophen on AKI appears unlikely but possible in a larger study with more AKI. We did confirm the strong association between intraoperative free hemoglobin concentrations and postoperative AKI that has been observed in prior studies.[1,3,4,6]

Taken together, the results of this study suggest that 1) although acetaminophen attenuates intraoperative plasma isofuran concentrations, the contribution of hemeprotein-mediated lipid peroxidation to systemic oxidative stress during CPB may be limited, 2) the effect of acetaminophen on hemeprotein-mediated lipid peroxidation may be obscured by plasma haptoglobin, and 3) free hemoglobin-mediated lipid peroxidation may play a minor role in the mechanism of postoperative AKI following CPB, particularly when plasma haptoglobin is not depleted. The protective role of haptoglobin is further supported by the observation that lower preoperative haptoglobin concentrations correlated with a higher incidence of postoperative AKI.

In summary, CPB is associated with hemoglobinemia, haptoglobinemia and lipid peroxidation. Perioperative intravenous acetaminophen attenuates the increase in intraoperative plasma isofuran concentrations but has no effect on urinary markers of lipid peroxidation. Although remote ischemic preconditioning reduces lipid peroxidation and has been employed to improve postoperative outcomes,[24] its effect on AKI is controversial. Some studies reported a protective effect whereas other studies showed no benefit.[25,26] To better define the role of hemeprotein-induced oxidative damage in cardiac surgery patients, future studies should investigate the use of ferryl protein reductants to inhibit hemeprotein-mediated lipid peroxidation during CPB and preserve postoperative kidney function.

## Supporting Information

S1 CONSORT Checklist CONSORT checklist. (DOC)

S1 Protocol Protocol for this trial. (DOC)

## References

[1] Mamikonian LS, Mamo LB, Smith PB, Koo J, Lodge AJ, et al (2014) Cardiopulmonary bypass is associated with hemolysis and acute kidney injury in neonates, infants, and children. Pediatr Crit Care Med 15: e111–119. 10.1097/PCC.0000000000000047 24394997 PMC3951557

[2] Vercaemst L (2008) Hemolysis in cardiac surgery patients undergoing cardiopulmonary bypass: a review in search of a treatment algorithm. J Extra Corpor Technol 40: 257–267. 19192755 PMC4680715

[3] Billings FT, Yu C, Byrne JG, Petracek MR, Pretorius M (2014) Heme oxygenase-1 and acute kidney injury following cardiac surgery. Cardiorenal Med 4: 12–21. 10.1159/000357871 24847330 PMC4024967

[4] Simpson SA, Zaccagni H, Bichell DP, Christian KG, Mettler BA, et al (2014) Acetaminophen attenuates lipid peroxidation in children undergoing cardiopulmonary bypass. Pediatr Crit Care Med 15: 503–510. 10.1097/PCC.0000000000000149 24732290 PMC4087071

[5] Boutaud O, Moore KP, Reeder BJ, Harry D, Howie AJ, et al (2010) Acetaminophen inhibits hemoprotein-catalyzed lipid peroxidation and attenuates rhabdomyolysis-induced renal failure. Proc Natl Acad Sci U S A 107: 2699–2704. 10.1073/pnas.0910174107 20133658 PMC2823910

[6] Billings FT 4th, Ball SK, Roberts LJ 2nd, Pretorius M (2011) Postoperative acute kidney injury is associated with hemoglobinemia and an enhanced oxidative stress response. Free Radic Biol Med 50: 1480–1487. 10.1016/j.freeradbiomed.2011.02.011 21334433 PMC3090463

[7] Billings FT, Pretorius M, Schildcrout JS, Mercaldo ND, Byrne JG, et al (2012) Obesity and oxidative stress predict AKI after cardiac surgery. J Am Soc Nephrol 23: 1221–1228. 10.1681/ASN.2011090940 22626819 PMC3380645

[8] Schulz KF, Altman DG, Moher D, Group C (2010) CONSORT 2010 statement: updated guidelines for reporting parallel group randomised trials. PLoS Med 7: e1000251 10.1371/journal.pmed.1000251 20352064 PMC2844794

[9] Balaguer JM, Yu C, Byrne JG, Ball SK, Petracek MR, et al (2013) Contribution of endogenous bradykinin to fibrinolysis, inflammation, and blood product transfusion following cardiac surgery: a randomized clinical trial. Clin Pharmacol Ther 93: 326–334. 10.1038/clpt.2012.249 23361105 PMC4031681

[10] Mehta RL, Kellum JA, Shah SV, Molitoris BA, Ronco C, et al (2007) Acute Kidney Injury Network: report of an initiative to improve outcomes in acute kidney injury. Crit Care 11: R31 17331245 10.1186/cc5713 PMC2206446

[11] Morgan CJ, Zappitelli M, Robertson CM, Alton GY, Sauve RS, et al (2013) Risk factors for and outcomes of acute kidney injury in neonates undergoing complex cardiac surgery. J Pediatr 162: 120–127 e121 10.1016/j.jpeds.2012.06.054 22878115

[12] Morrow JD (2005) Quantification of Isoprostanes as Indices of Oxidant Stress and the Risk of Atherosclerosis in Humans. Arterioscler Thromb Vasc Biol 25: 279–286. 15591226 10.1161/01.ATV.0000152605.64964.c0

[13] Fessel JP, Porter NA, Moore KP, Sheller JR, Roberts LJ (2002) Discovery of lipid peroxidation products formed in vivo with a substituted tetrahydrofuran ring (isofurans) that are favored by increased oxygen tension. Proc Natl Acad Sci U S A 99: 16713–16718. 12482927 10.1073/pnas.252649099 PMC139209

[14] Albers E, Donahue BS, Milne G, Saville BR, Wang W, et al (2012) Perioperative plasma F(2)-Isoprostane levels correlate with markers of impaired ventilation in infants with single-ventricle physiology undergoing stage 2 surgical palliation on the cardiopulmonary bypass. Pediatr Cardiol 33: 562–568. 10.1007/s00246-012-0166-2 22327227 PMC3641818

[15] Unger J, Filippi G, Patsch W (2007) Measurements of Free Hemoglobin and Hemolysis Index: EDTA- or Lithium-Heparinate Plasma? Clin Chem 53: 1717–1718. 17712012 10.1373/clinchem.2007.091421

[16] Milne GL, Sanchez SC, Musiek ES, Morrow JD (2007) Quantification of F2-isoprostanes as a biomarker of oxidative stress. Nat Protoc 2: 221–226. 17401357 10.1038/nprot.2006.375

[17] Davi G, Falco A, Patrono C (2005) Lipid peroxidation in diabetes mellitus. Antioxid Redox Signal 7: 256–268. 15650413 10.1089/ars.2005.7.256

[18] Reeder BJ, Svistunenko DA, Cooper CE, Wilson MT (2004) The radical and redox chemistry of myoglobin and hemoglobin: from in vitro studies to human pathology. Antioxid Redox Signal 6: 954–966. 15548893 10.1089/ars.2004.6.954

[19] Kato GJ (2009) Haptoglobin halts hemoglobins havoc. J Clin Invest 119: 2140–2142. 10.1172/JCI40258 19620777 PMC2719939

[20] Lim YK, Jenner A, Ali AB, Wang Y, Hsu SIH, et al (2000) Haptoglobin reduces renal oxidative DNA and tissue damage during phenylhydrazine-induced hemolysis. Kidney Int 58: 1033–1044. 10972668 10.1046/j.1523-1755.2000.00261.x

[21] Shin H, Yozu R, Maehara T, Matayoshi T, Morita M, et al (2000) Vacuum assisted cardiopulmonary bypass in minimally invasive cardiac surgery: its feasibility and effects on hemolysis. Artif Organs 24: 450–453. 10886064 10.1046/j.1525-1594.2000.06587.x

[22] Ulus AT, Aksoyek A, Ozkan M, Katircioglu SF, Basu S (2003) Cardiopulmonary bypass as a cause of free radical-induced oxidative stress and enhanced blood-borne isoprostanes in humans. Free Radic Biol Med 34: 911–917. 12654480 10.1016/s0891-5849(03)00030-3

[23] Alayash AI, Andersen CB, Moestrup SK, Bulow L (2013) Haptoglobin: the hemoglobin detoxifier in plasma. Trends Biotechnol 31: 2–3. 10.1016/j.tibtech.2012.10.003 23140673

[24] Li C, Xu M, Wu Y, Li YS, Huang WQ, et al (2014) Limb remote ischemic preconditioning attenuates lung injury after pulmonary resection under propofol-remifentanil anesthesia: a randomized controlled study. Anesthesiology 121: 249–259. 10.1097/ALN.0000000000000266 24743579

[25] Gallagher SM, Jones DA, Kapur A, Wragg A, Harwood SM, et al (2014) Remote ischemic preconditioning has a neutral effect on the incidence of kidney injury after coronary artery bypass graft surgery. Kidney Int. 10.1038/ki.2014.259 25075773

[26] Candilio L, Malik A, Ariti C, Barnard M, Di Salvo C, et al (2014) Effect of remote ischaemic preconditioning on clinical outcomes in patients undergoing cardiac bypass surgery: a randomised controlled clinical trial. Heart. 10.1136/heartjnl-2014-306178 25252696

[27] Levey AS, Stevens LA, Schmid CH, Zhang YL, Castro AF, et al (2009) A new equation to estimate glomerular filtration rate. Ann Intern Med 150: 604–612. 19414839 10.7326/0003-4819-150-9-200905050-00006 PMC2763564

