# Supplementary material for: Perioperative Intravenous Acetaminophen Attenuates Lipid Peroxidation in Adults Undergoing Cardiopulmonary Bypass: A Randomized Clinical Trial
Source: PLoS One. 2015 Feb 23;10(2):e0117625. doi: 10.1371/journal.pone.0117625 (PMC4338200; doi:10.1371/journal.pone.0117625)
Supplement: S1 Protocol — (DOC) [file pone.0117625.s002.doc]

Inhibition of Lipid Peroxidation during Cardiopulmonary Bypass

**Mias Pretorius, MBChB, MSCI**

561 PRB

Vanderbilt University Medical Center

Nashville, TN 37212

**Table of Contents:**

**Study Schema**

1. **Background**
2. **Rationale and Specific Aims**
3. **Animal Studies and Previous Human Studies**
4. **Inclusion/Exclusion Criteria**
5. **Enrollment/Randomization**
6. **Study Procedures**
7. **Risks of Investigational Agents/Devices (side effects)**
8. **Reporting of Adverse Events or Unanticipated Problems involving Risk to Participants or Others**
9. **Study Withdrawal/Discontinuation**
10. **Statistical Considerations**
11. **Privacy/Confidentiality Issues**
12. **Follow-up and Record Retention**
13. **Background**

Acute kidney injury (AKI) is a major complication of cardiac surgery requiring cardiopulmonary bypass (CPB). Hemolysis and rhabdomyolysis frequently occur during CPB. Hemolysis leads to an increase in free hemoglobin, whereas rhabdomyolysis leads to an increase in myoglobin. Free plasma hemoglobin and myoglobin have been shown to be independent predictors of the acute kidney injury that results from CPB. When these hemeproteins are released into the plasma, they undergo redox cycling, generating radical species that initiate lipid peroxidation and a cascade of oxidative damage to cellular membranes, notably in the kidney. F_2_-isoprostanes (F2-isoP) and isofurans (isoF) are sensitive and specific markers of oxidative stress *in vivo*, and are increased after CPB, particularly in those patients with AKI. Acetaminophen (ApAP) inhibits the lipid peroxidation catalyzed by myoglobin and hemoglobin. Moreover, in an animal model of rhabdomyolysis-induced kidney injury, ApAP significantly attenuated the decrease in creatinine clearance compared to control. The current proposal tests the central hypothesis that ApAP will attenuate the lipid peroxidation associated with the hemolysis and rhabdomyolysis that occur in patients undergoing CPB. Demonstration that ApAP inhibits the lipid peroxidation resulting from CPB would provide a rationale for a prospective randomized trial to test the hypothesis that ApAP will reduce the AKI that results from CPB. The duration of the study is 3 years.

1. **Rationale and Specific Aims**

**Specific Aims**

Many patients with heart disease require cardiac surgery involving cardiopulmonary bypass (CPB). Acute kidney injury (AKI) is a major complication of cardiac surgery following CPB and is an independent predictor of in-hospital mortality, morbidity, mid-term and long-term survival. ^1-6^ Thus far, no single strategy has demonstrated conclusively its ability to prevent AKI after CPB surgery.^6^ Concerted evidence supports a hypothesis that hemeprotein-catalyzed peroxidation of renal lipids is a major and potentially modifiable contributor to postoperative AKI. Hemolysis frequently occurs during CPB and is more pronounced in the pediatric surgical population.^7^ Hemolysis leads to an increase in free hemoglobin and is an independent predictor of postoperative AKI.^8^ When hemeproteins are released into the plasma, they undergo redox cycling, generating radical species that initiate lipid peroxidation and a cascade of oxidative damage to cellular membranes, notably in the kidney. ^9, 10^ F_2_-isoprostanes (F2-isoP) and isofurans (isoF) are sensitive and specific markers of oxidative stress *in vivo*,^11, 12^ and are increased after CPB, particularly in those patients with AKI (preliminary data). Analogous to its effect on the prostaglandin H synthases, acetaminophen (ApAP) reduces the ferryl oxo radical of hemeproteins and prevents formation of the amino acid radical, thus inhibiting hemeprotein-catalyzed lipid peroxidation.^9, 13^ Moreover, in an animal model of rhabdomyolysis-induced kidney injury, ApAP significantly attenuated the decrease in creatinine clearance compared to control.^9^ **The current proposal tests the central hypothesis that ApAP will attenuate the lipid peroxidation associated with the hemolysis that occurs in patients undergoing CPB.** Demonstration that ApAP inhibits the lipid peroxidation resulting from CPB would provide a rationale for a prospective randomized trial to test the hypothesis that ApAP will reduce AKI that results from CPB-induced hemolysis.

**Research Strategy**

**Significance**

Acute kidney injury (AKI) occurs in up to 30% of patients who undergo cardiac surgery and is an independent predictor of in-hospital mortality, morbidity, mid-term and long-term survival.^1-6^ Concerted evidence supports a hypothesis that hemeprotein-catalyzed peroxidation of renal lipids is a major and potentially modifiable contributor to the AKI. Plasma free hemoglobin and myoglobin are independent predictors of AKI in the early postoperative period. ^8, 14^ Hemolysis, a common consequence of CPB, results in increasing levels of plasma free hemoglobin and decreasing levels of haptoglobin.^7^ Maximal plasma free hemoglobin levels typically occurs at the end of bypass and fall slowly thereafter, reaching baseline after 24 hours.^15, 16^ Myoglobin concentrations also increase rapidly after CPB and indicates muscle injury.^14^ Free hemeproteins not only increase the consumption of nitric oxide, but also undergo hemeprotein redox cycling and increase lipid peroxidation.^9, 10, 17^ Therefore, hemeproteins can injure renal cells through various mechanisms including lipid peroxidation, vasoconstriction, and cast formation.^9, 18, 19^ Although various pharmacological strategies have been successful in attenuating hemeprotein-induced kidney injury in animal models, no single strategy has demonstrated conclusively its ability to prevent AKI after CPB surgery in humans.^6, 9, 20^ Despite efforts to reduce CPB-induced hemolysis (by improving circuit design, the use of centrifugal pumps^15^ or limiting the use of “pump suckers”) significant hemolysis still occurs and therefore alternative strategies are needed to attenuate hemeprotein-induced lipid peroxidation and potential renal injury.

We found increased levels of circulating free hemeproteins and increased lipid peroxidation in patients who developed AKI and correlations between levels of free hemoglobin, the extent of lipid peroxidation, and postoperative increases in levels of serum creatinine. (see PRELIMINARY STUDIES). These findings suggest that hemeprotein-induced oxidative injury contributes to postoperative AKI. Moreover, in an animal model of myoglobin-induced kidney failure ApAP (inhibitor of hemeprotein redox cycling) not only reduced markers of oxidative stress but also significantly attenuated the decrease in kidney function.^9^ Therefore, our preliminary data in humans and animals suggest the hypothesis that ApAP will attenuate the lipid peroxidation associated with the hemolysis that occurs in patients undergoing CPB.

F2-isoP and isoF, formed by non-enzymatic free radical-induced peroxidation of arachidonic acid, are sensitive and specific markers of oxidative stress and have emerged as the “gold standard” assessment of oxidative stress *in vivo*.^11, 12^ Both F2-isoP and isoF increase significantly during CPB (see PRELIMINARY STUDIES, Figure 1A). F2-isoPs are not only markers of oxidative stress but have been shown to be powerful vasoconstrictors and contribute to the pathogenesis and organ dysfunction associated with rhabdomyolysis, subarachnoid hemorrhage and hemolytic disorders.^9, 21-23^ As oxygen tension increases, the formation of F2-isoPs become disfavored whereas the formation of isoFs increase.^12^ During CPB surgery higher oxygen tensions favors the formation of isoF (see PRELIMINARY STUDIES, Figure 1B). Thus, combined measurement of both F2-isoP and isoF afford the most reliable approach to assess oxidative stress.

Although the pathophysiology of CPB-induced AKI is certainly multifactorial,^4, 24-27^ this research addresses the hypothesis that hemeprotein-catalyzed lipid peroxidation in the kidney is a major contributor, and that is one that can be ameliorated pharmacologically. This hypothesis is based on the documented release of hemoglobin and myoglobin during CPB, the association of the levels of these hemeproteins with AKI, our novel evidence linking lipid peroxidation with AKI, and the abundant evidence that hemeprotein-catalyzed lipid peroxidation can cause AKI. This proof of concept study will test the hypothesis that ApAP inhibits hemeprotein redox cycling induced oxidative stress in patients undergoing CPB. If ApAP attenuates the oxidative stress response then a larger clinical trial will be designed with AKI as the primary outcome.

**Innovation**

CPB causes hemolysis and oxidative stress that may contribute to kidney dysfunction.^8, 16^ Thus far, no single strategy has demonstrated conclusively its ability to prevent AKI after CPB surgery and therefore alternative strategies are needed.^6^This study is novel in that it will evaluate the antioxidant effect of ApAP on hemeprotein-induced oxidative stress. If ApAP attenuates the oxidative stress response associated with CPB-induced hemolysis, we will design a larger prospective randomized trial to test the hypothesis that ApAP will reduce AKI associated with hemoprotein-induced oxidative stress following CPB. We will use the most reliable *in vivo* markers of oxidative stress to assess the correlation between oxidative stress and CPB-associated hemolysis and the efficacy of ApAP.^28^

**Rationale**

CPB induces hemolysis, rhabdomyolysis and oxidative stress that may contribute to AKI in patients undergoing cardiac surgery. ApAP inhibits hemeprotein-induced oxidative stress in an animal model.^9^ This study will test the hypothesis that ApAP attenuates the oxidative stress response, as measured by F_2_-IsoP and isoFs in adults undergoing cardiac surgery requiring CPB.

Subjects

Fifty-two subjects will be studied in a placebo-controlled 2-arm design. Patients will be eligible if they are undergoing elective cardiac surgery requiring CPB. Patients will be excluded if they are undergoing off-pump surgery, undergoing emergency surgery, presence of cardiogenic shock, or have renal insufficiency (baseline creatinine >2 mg/dl).

Standard techniques

*Instrumentation and CPB*

After induction of anesthesia all subjects will receive, a radial or femoral arterial line, a central venous line and a pulmonary artery catheter. All the above monitoring is part of the routine care of a patient undergoing cardiac surgery. All blood will be drawn through the central venous line. Anesthesia management and CPB will be conducted according to institutional protocol. Patients will receive general endotracheal anesthesia. CPB will be achieved with a roller or centrifugal pump (Medtronic, Minneapolis, MN), heparin coated circuit (Carmeda) and a Trillium hollow fiber oxygenator (Medtronic, Minneapolis, MN). Heparin will be used for anticoagulation during CPB at an initial dose of 400 U/kg supplemented with additional heparin to achieve and maintain an activated clotting time (ACT) of >400s. Heparin will be neutralized with protamine sulfate, after separation from CPB.

Anticipated Results

Based on our preliminary data we anticipate that CPB will increase oxidative stress as measured by F_2_-IsoP and isoFs and that there will be a correlation between F_2_-isoprostane and isoF concentrations and hemoprotein concentrations in the placebo group. We further anticipate that ApAP will attenuate the increase in F_2_-isoprostane and isoF concentrations compared to placebo. In addition, we anticipate that ApAP will decrease markers of AKI (NGAL) compared to placebo.

Potential Limitations and Future Directions

This study will provide important data concerning the potential protective effect of ApAP on hemeprotein-induced AKI in patients undergoing CPB. If ApAP significantly reduces oxidative stress and markers of AKI, we plan to design a prospective double-blinded randomized clinical trial to test the hypothesis that ApAP reduces AKI in patients undergoing CPB.

Timeline

Vanderbilt University Medical Center performs on average 1200 cardiac surgeries annually of which approximately 600 are on-pump. We anticipate enrolling the 52 study patients within the first 2 ½ years of the study (21 patients per year). The last 6 months will be used for data analysis and manuscript writing. We have the full support from the cardiothoracic surgeons.

Assays

All blood samples drawn for research assays will be centrifuged for 20 minutes immediately after blood drawing, and plasma or serum will labeled with the case identification number and stored at -80°C until sampling. Urine collections of 5 ml will be collected from a Foley catheter placed as part of cardiac surgery protocol. Urine will be processed promptly by centrifuge, with supernatant frozen and stored at -80°C. Urine and plasma F_2_-IsoP and isoFs will be measured using stable isotope dilution methods in conjunction with gas chromatography and mass spectrometry as previously described^29, 30^ Haptoglobin will be measured by an immunoturbidimetric method to assess the severity of intravascular hemolysis. Free hemoglobin concentrations will be spectrophotmetrically determined by using the difference of absorbances at 540 and 600nm as described by Unger et al.^31^ Myoglobin will be assayed with a commercially available ELISA kit. ApAP plasma levels will be assayed using a commercially available enzymatic/color method to determine plasma levels obtained during CPB and on POD1. NGAL, an early marker for AKI,^32^ will be assayed using a commercially available ELISA kit.

**References**

**1.** Karkouti K, Wijeysundera DN, Yau TM, Callum JL, Cheng DC, Crowther M, Dupuis J-Y, Fremes SE, Kent B, Laflamme C, Lamy A, Legare J-F, Mazer CD, McCluskey SA, Rubens FD, Sawchuk C, Beattie WS. Acute kidney injury after cardiac surgery: focus on modifiable risk factors. *Circulation.* 2009;119:495-502.

**2.** Billings F, Siew E, Yu C, Pretorius M, Brown N. Early postoperative statin therapy is associated with a lower incidence of acute kidney injury following cardiac surgery. *J Cardiothorac Vasc Anesth.* 2010;24:913-920.

**3.** Mangano CM, Diamondstone LS, Ramsay JG, Aggarwal A, Herskowitz A, Mangano DT. Renal Dysfunction after Myocardial Revascularization: Risk Factors, Adverse Outcomes, and Hospital Resource Utilization. *Ann Intern Med.* 1998;128:194-203.

**4.** Zanardo G, Michielon P, Paccagnella A, Rosi P, Calo M, Salandin V, Da Ros A, Michieletto F, Simini G. Acute renal failure in the patient undergoing cardiac operation. Prevalence, mortality rate, and main risk factors. *J Thorac Cardiovasc Surg.* 1994;107:1489-1495.

**5.** Chertow GM, Levy EM, Hammermeister KE, Grover F, Daley J. Independent association between acute renal failure and mortality following cardiac surgery. *Am J Med.* 1998;104:343-348.

**6.** Rosner MH, Okusa MD. Acute kidney injury associated with cardiac surgery. *Clin J Am Soc Nephrol.* 2006;1:19-32.

**7.** Vercaemst L. Hemolysis in cardiac surgery patients undergoing cardiopulmonary bypass: a review in search of a treatment algorithm. *J Extra Corpor Technol.* 2008;40:257-267.

**8.** Vermeulen Windsant IC, Snoeijs MG, Hanssen SJ, Altintas S, Heijmans JH, Koeppel TA, Schurink GW, Buurman WA, Jacobs MJ. Hemolysis is associated with acute kidney injury during major aortic surgery. *Kidney Int.* 2010;77:913-920.

**9.** Boutaud O, Moore KP, Reeder BJ, Harry D, Howie AJ, Wang S, Carney CK, Masterson TS, Amin T, Wright DW, Wilson MT, Oates JA, Roberts LJ, 2nd. Acetaminophen inhibits hemoprotein-catalyzed lipid peroxidation and attenuates rhabdomyolysis-induced renal failure. *Proc Natl Acad Sci U S A.* 2010;107:2699-2704.

**10.** Reeder BJ, Svistunenko DA, Cooper CE, Wilson MT. The radical and redox chemistry of myoglobin and hemoglobin: from in vitro studies to human pathology. *Antioxid Redox Signal.* 2004;6:954-966.

**11.** Morrow JD. Quantification of Isoprostanes as Indices of Oxidant Stress and the Risk of Atherosclerosis in Humans. *Arterioscler Thromb Vasc Biol.* 2005;25:279-286.

**12.** Roberts LJ, 2nd, Fessel JP. The biochemistry of the isoprostane, neuroprostane, and isofuran pathways of lipid peroxidation. *Chem Phys Lipids.* 2004;128:173-186.

**13.** Aronoff DM, Oates JA, Boutaud O. New insights into the mechanism of action of acetaminophen: Its clinical pharmacologic characteristics reflect its inhibition of the two prostaglandin H2 synthases. *Clin Pharmacol Ther.* 2006;79:9-19.

**14.** Benedetto U, Angeloni E, Luciani R, Refice S, Stefanelli M, Comito C, Roscitano A, Sinatra R. Acute kidney injury after coronary artery bypass grafting: does rhabdomyolysis play a role? *J Thorac Cardiovasc Surg.* 2010;140:464-470.

**15.** Morgan IS, Codispoti M, Sanger K, Mankad PS. Superiority of centrifugal pump over roller pump in paediatric cardiac surgery: prospective randomised trial. *Eur J Cardiothorac Surg.* 1998;13:526-532.

**16.** Christen S, Finckh B, Lykkesfeldt J, Gessler P, Frese-Schaper M, Nielsen P, Schmid ER, Schmitt B. Oxidative stress precedes peak systemic inflammatory response in pediatric patients undergoing cardiopulmonary bypass operation. *Free Radic Biol Med.* 2005;38:1323-1332.

**17.** Rother RP, Bell L, Hillmen P, Gladwin MT. The Clinical Sequelae of Intravascular Hemolysis and Extracellular Plasma Hemoglobin: A Novel Mechanism of Human Disease. *Jama.* 2005;293:1653-1662.

**18.** Nath KA, Grande JP, Croatt AJ, Likely S, Hebbel RP, Enright H. Intracellular targets in heme protein-induced renal injury. *Kidney Int.* 1998;53:100-111.

**19.** Zager RA, Gamelin LM. Pathogenetic mechanisms in experimental hemoglobinuric acute renal failure. *Am J Physiol.* 1989;256:F446-455.

**20.** Plotnikov EY, Chupyrkina AA, Pevzner IB, Isaev NK, Zorov DB. Myoglobin causes oxidative stress, increase of NO production and dysfunction of kidney's mitochondria. *Biochim Biophys Acta.* 2009;1792:796-803.

**21.** Holt S, Reeder B, Wilson M, Harvey S, Morrow JD, Roberts LJ, 2nd, Moore K. Increased lipid peroxidation in patients with rhabdomyolysis. *Lancet.* 1999;353:1241.

**22.** Lin CL, Hsu YT, Lin TK, Morrow JD, Hsu JC, Hsu YH, Hsieh TC, Tsay PK, Yen HC. Increased levels of F2-isoprostanes following aneurysmal subarachnoid hemorrhage in humans. *Free Radic Biol Med.* 2006;40:1466-1473.

**23.** Klings ES, Christman BW, McClung J, Stucchi AF, McMahon L, Brauer M, Farber HW. Increased F2 isoprostanes in the acute chest syndrome of sickle cell disease as a marker of oxidative stress. *Am J Respir Crit Care Med.* 2001;164:1248-1252.

**24.** Mack MJ, Pfister A, Bachand D, Emery R, Magee MJ, Connolly M, Subramanian V. Comparison of coronary bypass surgery with and without cardiopulmonary bypass in patients with multivessel disease. *J Thorac Cardiovasc Surg.* 2004;127:167-173.

**25.** Scolari F, Tardanico R, Zani R, Pola A, Viola BF, Movilli E, Maiorca R. Cholesterol crystal embolism: A recognizable cause of renal disease. *Am J Kidney Dis.* 2000;36:1089-1109.

**26.** Boldt J, Brenner T, Lehmann A, Suttner SW, Kumle B, Isgro F. Is kidney function altered by the duration of cardiopulmonary bypass? *Ann Thorac Surg.* 2003;75:906-912.

**27.** Miller CC, 3rd, Villa MA, Sutton J, Lau D, Keyhani K, Estrera AL, Azizzadeh A, Coogan SM, Safi HJ. Serum myoglobin and renal morbidity and mortality following thoracic and thoraco-abdominal aortic repair: does rhabdomyolysis play a role? *Eur J Vasc Endovasc Surg.* 2009;37:388-394.

**28.** Kadiiska MB, Gladen BC, Baird DD, Germolec D, Graham LB, Parker CE, Nyska A, Wachsman JT, Ames BN, Basu S, Brot N, Fitzgerald GA, Floyd RA, George M, Heinecke JW, Hatch GE, Hensley K, Lawson JA, Marnett LJ, Morrow JD, Murray DM, Plastaras J, Roberts LJ, 2nd, Rokach J, Shigenaga MK, Sohal RS, Sun J, Tice RR, Van Thiel DH, Wellner D, Walter PB, Tomer KB, Mason RP, Barrett JC. Biomarkers of oxidative stress study II: are oxidation products of lipids, proteins, and DNA markers of CCl4 poisoning? *Free Radic Biol Med.* 2005;38:698-710.

**29.** Milne GL, Sanchez SC, Musiek ES, Morrow JD. Quantification of F2-isoprostanes as a biomarker of oxidative stress. *Nat Protoc.* 2007;2:221-226.

**30.** Milne GL, Yin H, Brooks JD, Sanchez S, Jackson Roberts L, 2nd, Morrow JD. Quantification of F2-isoprostanes in biological fluids and tissues as a measure of oxidant stress. *Methods Enzymol.* 2007;433:113-126.

**31.** Unger J, Filippi G, Patsch W. Measurements of Free Hemoglobin and Hemolysis Index: EDTA- or Lithium-Heparinate Plasma? *Clin Chem.* 2007;53:1717-1718.

**32.** McIlroy DR, Wagener G, Lee HT. Biomarkers of Acute Kidney Injury: An Evolving Domain. *Anesthesiology.* 2010;112:998-1004.

**33.** Bannwarth B, Pehourcq F. [Pharmacologic basis for using paracetamol: pharmacokinetic and pharmacodynamic issues]. *Drugs.* 2003;63 Spec No 2:5-13.

**34.** Lonnqvist PA, Morton NS. Postoperative analgesia in infants and children. *Br J Anaesth.* 2005;95:59-68.

**35.** Ulus AT, Aksoyek A, Ozkan M, Katircioglu SF, Basu S. Cardiopulmonary bypass as a cause of free radical-induced oxidative stress and enhanced blood-borne isoprostanes in humans. *Free Radic Biol Med.* 2003;34:911-917.

**36.** Diggle PJ, Heagerty P, Liang KY, Zeger SL. *Analysis of Longitudinal Data*. 2nd ed: Oxford University Press; 2002.

1. **Animal Studies and Previous Human Studies**

Preliminary Human Studies

Oxidative stress and hemolysis are increased in patients who developed AKI following CPB. In a nested case-control study of 20 adult patients undergoing CPB, we compared markers of oxidative stress and hemolysis in 10 AKI patients with 10 controls (No AKI). Markers of oxidative stress (F2-isoP and isoF concentrations), and hemolysis (plasma free hemoglobin) were measured at baseline before surgery, throughout the intraoperative period and on postoperative day (POD) 1-3. AKI was defined as an increase in serum creatinine >0.3mg/dl from baseline within 48hrs after surgery. CPB caused hemolysis with a significant increase in free hemoglobin (P<0.001). Peak free hemoglobin concentrations occurred post CPB and returned to baseline on POD1. Peak free hemoglobin concentrations were significantly greater in patients who developed AKI compared to controls (289.0±37.8 versus 104.4±36.5 mg/dl, P=0.01). Plasma concentrations of both F2-isoP and isoF increased significantly during CPB and returned to baseline on POD1 (P=0.004, P=0.001 for effect of time respectively). Baseline F2-isoP concentrations were significantly higher compared to baseline isoF concentrations (46.1±4.2 versus 32.5±4.21 pg/ml, P=0.01). The magnitude in increase, as measured by the percentage change from baseline, was significantly greater for isoF compared to F2-isoP in plasma (ANOVA P=0.02, Figure 1A) and in urine (ANOVA P=0.01, Figure 1B) Baseline isoF concentrations tended to be higher in the control group compared to the AKI group (38.2±6.3 versus 27.4±5.4 pg/ml, P=0.07). Because of these differences in baseline markers of oxidative stress we calculated the percentage change from baseline to compare the oxidative stress response between the two groups. The percentage change from baseline for both plasma F2-isoP and plasma isoF (Figure 2A) were significantly higher in the AKI group compared to the control group (P=0.02 and P=0.01 respectively). Although the early increase in urine isoF concentrations was similar between study groups (P=0.97) there was a sharp divergence in response following surgery such that urine isoF concentrations continued to rise, peaked on postoperative day 1, and remained elevated on postoperative day 2 in the AKI group (P=0.04, Figure 2B). In addition, change in peak plasma isoF concentrations correlated not only with peak free plasma hemoglobin concentrations (r^2^=0.39, P=0.001) but also with change in peak serum creatinine (r^2^=0.20, P=0.01). **These results suggest that 1) both F2-isoP and isoF concentrations increase significantly during CPB and may be useful biomarkers to assess the oxidative stress response in patients undergoing CPB, 2) the change in isoF concentrations were greater compared to the change in F2-isoP concentrations and may reflect the role of an increased oxygen partial pressure during surgery, 3) postoperative AKI is associated with both enhanced intraoperative hemeprotein release and enhanced lipid peroxidation, and 4) the correlations among hemoglobinemia, lipid peroxidation and kidney injury indicate a potential role of hemeprotein redox cycling induced oxidative stress in the pathogenesis of postoperative AKI.** (These results are now in press. Please see attached manuscript).

1. **Inclusion/Exclusion Criteria**

*Inclusion Criteria*

1. Subjects, 18 to 80 years of age, scheduled for elective cardiac surgery requiring CPB
2. For female subjects, the following conditions must be met:

postmenopausal for at least 1 year, or

status-post surgical sterilization, or

if of childbearing potential, utilizing adequate birth control and a negative urine beta-hcg prior to drug treatment

*Exclusion Criteria*

1. Allergic reaction to ApAP (acetaminophen)
2. Evidence of severe hepatic impairment (history of liver cirrhosis or total bilirubin >2.0mg/dl
3. Impaired renal function (serum creatinine >2.0 mg/dl)
4. Emergency surgery
5. Pregnancy
6. Breast-feeding
7. Any underlying or acute disease requiring regular medication which could possibly pose a threat to the subject or make implementation of the protocol or interpretation of the study results difficult
8. History of alcohol or drug abuse
9. Treatment with any investigational drug in the 1 month preceding the study
10. Mental conditions rendering the subject unable to understand the nature, scope and possible consequences of the study.
11. Inability to comply with the protocol, e.g. uncooperative attitude and unlikelihood of completing the study
12. **Enrollment/Randomization**

*Subject Selection*

Fifty-two subjects will be studied in a placebo-controlled 2-arm design. Patients will be eligible if they are undergoing elective cardiac surgery requiring CPB. Patients will be excluded if they are undergoing off-pump surgery, undergoing emergency surgery, presence of cardiogenic shock, or have renal insufficiency (baseline creatinine >2 mg/dl)

On the day of surgery patients will be instrumented and subjected to CPB as described under STANDARD TECHNIQUES. All existing preoperative medications will be continued until the day of surgery. Patients will be instructed not to take any ApAP within 24 hours prior to surgery.

a. *Source of Drugs*

Patients will be randomized to receive ApAP or placebo shortly after intubation in the OR but before CPB. The Vanderbilt Investigational Pharmacy will be responsible for the storage, preparation, and labeling of all investigational agents. The Clinical Research Pharmacist will devise standard operating procedures for the pharmacy to follow with regard to preparing, labeling, blinding and dispensing study drug. The investigational pharmacy is also responsible for maintaining accurate drug storage and dispensing logs.

b. *Randomization and Drug Treatment*

All drug administration will be double-blind. Subjects will be randomly assigned to treatment using a permuted-block randomization algorithm. Intravenous ApAP (OFIRMEV) will be given at a standard dose of 1g every 6 hours for a weight >50kg (maximum of 4g per 24 hours) or 15mg/kg every 6 hours for a weight <50kg (maximum of 75mg/kg per 24 hours) starting shortly after intubation in the OR and before the start of CPB for a total of four doses. ApAP inhibits oxidation of arachidonic acid catalyzed by hemoglobin with an IC_50_ of 17.7±2.5μM which is well within the therapeutic range for humans (10-30μg/mL; 67-200μM).^9^ ApAP plasma concentrations will be measured to assess plasma concentrations achieved during and after CPB. The duration of study drug treatment is 24 hours since both free hemoglobin and markers of oxidative stress return to baseline within 24 hours. ^15, 16, 35^ While the patient is receiving study drug (24 hour period) no therapeutic ApAP will be allowed to be administered. After the first 24 hour period open label ApAP may be administered if clinically indicated.

1. **Study Procedures**

*Randomization and Drug Treatment*

Subjects will be randomly assigned to treatment using a permuted-block randomization algorithm. This 2-arm study will randomize patients to either ApAP or placebo. ApAP will be given at a standard dose of 1g every 6 hours for a weight >50kg (maximum of 4g per 24 hours) or 15mg/kg every 6 hours for a weight <50kg (maximum of 75mg/kg per 24 hours) for a total of 4 doses.

*Blood and Urine Sampling*

Blood and urine samples will be obtained for measurement of F_2_-IsoP, F2-isoF, haptoglobin, free hemoglobin, myoglobin, ApAP, and neutrophil gelatinase associated lipocalin (NGAL) from subjects undergoing CPB. Blood and urine samples will be taken at 6 different time points. The first sample is taken in the operating room before the start of CPB. Samples will then be drawn after 30 and 60 minutes on CPB, at the conclusion of CPB and upon arrival in the ICU. Finally, a postoperative sample will be collected on postoperative day 1 (POD1) for a total of approximately 135ml of blood (5 ounces) and 45ml of urine. Not every marker will be collected at every blood draw (Table).

| Blood/Urine Test | Pre CPB | 30min | 60min | Post-protamine | ICU | POD1 |
| --- | --- | --- | --- | --- | --- | --- |
| Plasma F2-IsoP/isofurans | √ | √ | √ | √ | √ | √ |
| Haptoglobin | √ | √ | √ | √ | √ | √ |
| Free Hemoglobin | √ | √ | √ | √ | √ | √ |
| Myoglobin | √ | √ | √ | √ | √ | √ |
| ApAP |  |  | √ | √ |  | √ |
| Urine F2-IsoP /isofurans | √ | √ | √ | √ | √ | √ |
| Urine NGAL | √ |  |  | √ |  | √ |

1. **Risks of Investigational Agents/Devices (side effects)**

**Potential Risks**

1. Insertion of venous catheters may cause bleeding, bruising, or infection- part of standard care
2. Frequent blood drawing can lead to anemia
3. Overdose of ApAP can cause liver damage (rare occurrence)

**Adequacy of protection against risks**

***Recruitment and Informed Consent***

We have prior experience recruiting adults (**ClinicalTrials.gov Identifier: NCT00223704**) as well as children undergoing cardiac surgery (**ClinicalTrials.gov Identifier: NCT00848250**). All cardiac surgery patients scheduled will be screened for eligibility. Surgical schedules are sent out each week to the cardiothoracic surgeons and cardiologists. Once identified, patients will be approached for consent during the following times; 1) preoperative cardiothoracic clinic visit, 2) immediately prior to surgery for cases admitted to Vanderbilt University Medical Center and requiring cardiac surgery within hours/days of admission, 3) in-patients at Vanderbilt University Medical Center who are monitored prior to surgery.

***Protections against Risk***

If a patient develops a fever preoperatively, he/she will be instructed to contact the surgical team and the case will be reviewed by the Anesthesia and Cardiothoracic teams. Most likely, the surgery will be postponed to a later date, to reduce the risk of pre-operative infection and complications. Patients will be instructed not to take any ApAP within 24 hours prior to surgery. If a patient develops a fever in the 1^st^ 24 hours (an event with a low risk of occurrence) following surgery, and has no significant bleeding, ibuprofen will be given for fever control. ApAP or placebo will be given at a standard dose (1g every 6 hours for 4 doses if weight is >50kg or 15mg/kg every 6 hours if weight is <50kg) over a 24 hour period. **To prevent overdosing, no additional ApAP (open label) will be allowed during the 24 hour study period.** We have designed the study to minimize the risk of liver injury by 1) excluding patients with a history of chronic liver disease, 2) excluding patients with a history of alcohol or drug abuse 3) limiting the dose of ApAP to 4g/24 hours for a weight > 50kg or 75mg/kg per 24 hours if weight is <50kg, 4) limiting the duration of study drug administration to 24 hours, and 5) utilizing WizOrder computerized entry to prevent the administration of open label ApAP during the 24 hour study period.

Several procedures will be implemented to improve adherence to the study protocol. We will provide training for the Anesthesia and ICU nursing staff concerning the study protocol prior to study implementation. In addition, we will utilize WizOrder (a computerized provider order entry (CPOE) system) to enter the study protocol and provide guidelines for study drug administration. WizOrder is Vanderbilt University Medical Center CPOE system with integrated decision support. Currently, all inpatient orders are entered using WizOrder. The CPOE system features a programmable rules engine used to deliver web-based decision support modules for the implementation of guidelines/protocols.

There are many safeguards in place to prevent the release of information from this study. All research samples obtained for this study will be assigned a code. The key to the code will be kept in a locked file in the investigator’s office. Only the investigator, co-investigators and research nurse will have access to the code and information that identifies subjects as being in this study. The results of tests run on research samples will not be recorded in subject’s medical records and neither the subject nor his doctor will be told of the results.

***Data and Safety Monitoring Plan***

All protocols will be reviewed and approved by the Vanderbilt University Institutional Review Board before subjects are enrolled. The Principal Investigator will closely oversee the protocol in conjunction with a dedicated research nurse (Patricia Wright, RN) to monitor for any adverse events or toxicities. Any adverse events or toxicities will be reported as per IRB guidelines. Patients will be monitored continuously throughout surgical intervention while on CPB and during postoperative care provided in the critical care unit.

Electronic data have several levels of protection. Any file that may contain patient identifiers will be password protected. All computers upon which data are stored are likewise password protected and are running anti-virus programs that automatically update definitions. All data are automatically backed-up to a secondary hard drive on a nightly basis. Access to the Vanderbilt computer network is protected at the level of firewalls, TCP wrappers and university assigned user ID’s. Data are secured with encryption algorithms and the network is maintained by the Medical Center’s Network Computer Services.

***Data management and quality control***

An electronic data collection form will be designed to minimize missing and erroneous values. This form will be pilot-tested before use. Data will be input into a protected web-based case report form (which can be readily downloaded into an SPSS or other database spreadsheet). The form allows for direct data entry by investigators. The program includes a computerized audit trail so that the identity of individuals entering or changing data and, in the case of changes, both original and revised data are saved. Data are backed up daily. Clinical data, including clinical laboratory, will be entered by the research nurse at Vanderbilt University Medical Center. Research laboratory data will be entered by Mr. Jeff Petro. A unique identification case number will be used to protect the confidentiality of the study participants. The case numbers and participants' names will be included in the protected source database but only case numbers will be included in any spreadsheet used for the statistical analysis. Before analysis, a senior research fellow in Dr. Pretorius’ laboratory, Dr. Hayden Zaccagni, will independently and blindly assess all raw data for accuracy and completeness. Dr. Yu Shyr, biostatistician, will check for potential outliers and resolve them with the investigators before unblinding the data and performing statistical analyses with input from Dr. Pretorius based on the methods specified in the data analysis plan. Prior to implementing the study protocol we will meet with all cardiac surgeons, cardiac anesthesiologists, critical care physicians and ICU nursing staff to provide training and information regarding the study design and administration of the study drug.

***Extracting clinical data***

The Data Transfer Service (DTS) feature of REDCap will be used to extract clinical data from the participant’s electronic medical record.  DTS will create a link between the participant’s electronic medical record and REDCap to pull study required data (e.g. laboratory values) for the study staff to review and include as part of the research record.   Access to adjudication screens for data pulls will be limited to individuals who are known to have access rights for StarPanel.  Data transfer services depend on medical record number and dates of service so that the linkage will have no significant value following study finalization.     All DTS functions are data extraction/transfer are logged with date-time stamp and user identification.

1. **Reporting of Adverse Events or Unanticipated Problems involving Risk to Participants or Others**

The Principal Investigator will closely oversee the protocol in conjunction with a dedicated research nurse (Patricia Wright, RN) to monitor for any adverse events or toxicities. Any adverse events or toxicities will be reported as per IRB guidelines. The investigators, the research coordinator, and the research nursing staff will monitor the patients daily for the purpose of tracking adverse events (AE). The research team will be informed of the participants’ progress. During the conduct of the trial, any serious adverse event (SAE) will be reported to the IRB within 24 hours. A SAE is any untoward medical occurrence that a) results in death, b) is life-threatening, c) requires prolongation of existing hospitalization, d) results in persistent or significant disability/incapacity. AE's will be graded as Mild (no limitation of usual activities), Moderate (some limitation) or Severe (inability to carry out usual activities) and attributed according to the relationship to the study drug and/or procedures as Not related, Unlikely, Possible, Probable, or Definite. This study is a pilot study using a FDA approved drug in standard dosages, frequency and route of administration and therefore a DSMB was not assembled. However, if the need should arise in the event of a SAE that is thought to be related to study drug then an ad hoc DSMB will be assembled to review the SAE and make a recommendation to the IRB.

1. **Study Withdrawal/Discontinuation**

This is an in-patient study. The patient can withdraw from the study at any time. Study drug will be discontinued in the event of liver injury (elevated liver enzymes). We have, however, designed the study to minimize the risk of liver injury by 1) excluding patients with a history of chronic liver disease, 2) excluding patients with a history of alcohol or drug abuse 3) limiting the dose of ApAP to 4g/24 hours for a weight > 50kg or 75mg/kg per 24 hours if weight is <50kg, 4) limiting the duration of study drug administration to 24 hours, and 5) utilizing WizOrder computerized entry to prevent the administration of open label ApAP during the 24 hour study period.

1. **Statistical Considerations**

*Primary and Secondary Outcomes*

The primary outcome is the oxidative stress response as measured by F_2_-IsoP and isoF concentrations. Because free hemoglobin (hemolysis) and myoglobin (rhabdomyolysis) have been associated with AKI we will assess renal function as a secondary outcome in the immediate postoperative period. To assess renal function we will collect already available data including urine output, BUN/Creatinine and daily fluid ins and outs. Other potential confounders of AKI including CPB time, daily use of vasopressors and re-exploration for bleeding will be collected. In addition, we will also measure urine NGAL as an early marker for AKI.

*Statistical Analysis and Power Calculation*

The primary objective of the analyses will be:

1. To compare the effect of ApAP versus placebo on the F_2_-isoprostane and isoF response to CPB

*Sample Size and Power Calculation*

Sample size calculations were based on CPB-induced increase in isoF concentrations based on preliminary data; plasma isoF concentrations increased from 32.5 (SD=17.9) to a peak of 57.6 (SD=25) pg/ml. In an animal study by Boutaud et al.^9^ ApAP reduced plasma F_2_-IsoP concentrations by approximately 40%. We assume that ApAP will reduce peak plasma isoF concentrations to a similar degree in humans. A sample size of 22 in each group will have 85% power to detect a difference in means of 23.1 (the difference between the placebo group mean, m_1_, of 57.6 and the ApAP group mean, m_2_, of 34.5) assuming that the within group standard deviation is 25 using a two group Satterthwaite t-test with a 0.05 two-sided significance level. If the reduction is 35% the study will still have 78% power assuming similar coefficient of variation in the two groups. If we further assumed that combined patient dropout and/or incomplete data sets have an occurrence rate of up to 20%, we determined that enrollment of 52 subjects (26 per group) would be required for this study.

*Data Analysis Plan*

We will use strict data quality control procedures (see Protection of Human Subjects). Standard graphing and screening techniques will be used to detect outliers and to ensure data accuracy. Summary statistics for both continuous and categorical variables will be provided by study arms to describe the study sample. Comparability of potential confounding factors as well as other baseline factors between the two randomization groups will be assessed as follows. Fisher's exact test will be used to assess categorical variables between groups. Differences between group means for continuous variables will be examined using Wilcoxon rank sum test.

The primary endpoint is peak F_2_-isoprostane and isoF concentrations. The between-group difference will be examined using t-test and Wilcoxon rank sum test. The difference will be estimated and reported along with a 95% confidence interval. F_2_-IsoP and isoFs will also be measured repeatedly at baseline, 30min, 60min, post-protamine, ICU and on POD 1. We will fit a mixed effect model for the longitudinal measures of F_2_-IsoP and isoFs with fixed effects of ApAP treatment (yes, no) and time since randomization. We will explore different plausible covariance matrix structures for F2-IsoP and isoFs, such as compound symmetry and a first-order autoregressive process [AR(1)], in the mixed effect model. We will check the model fitting by superimposing the fitted mean response profiles on a time-plot of the average observed response within each combination of the treatment and time, and to superimpose the fitted variogram on a plot of the empirical variogram^36^ The secondary endpoints include BUN, creatinine, and urine NGAL. These endpoints will be analyzed similarly as F2-IsoP and isoFs. This is an in-patient study. Once a patient consents to participate in the study, the chance for missing data is small. Every effort will be made to avoid missing data and we expect the impact of missing data will be minimal. The mixed effect model can also accommodate missing data at specific time points without excluding data from other completed time points. The statistical hypotheses will be tested at the level of α=0.05. This data analysis plan will be carried out using statistical software SPSS for Windows (Version 17.0, SPSS, Chicago) and R software (r-project.org).

1. **Privacy/Confidentiality Issues**

There are many safeguards in place to prevent the release of information from this study. All research samples obtained for this study will be assigned a code. The key to the code will be kept in a locked file in the investigator’s office. Only the investigator, co-investigators and research nurse will have access to the code and information that identifies subjects as being in this study. The results of tests run on research samples will not be recorded in subject’s medical records and neither the subject nor his doctor will be told of the results. Electronic data have several levels of protection. Any file that may contain patient identifiers will be password protected. All computers upon which data are stored are likewise password protected and are running anti-virus programs that automatically update definitions. All data are automatically backed-up to a secondary hard drive on a nightly basis. Access to the Vanderbilt computer network is protected at the level of firewalls, TCP wrappers and university assigned user ID’s. Data are secured with encryption algorithms and the network is maintained by the Medical Center’s Network Computer Services.

1. **Follow-up and Record Retention**

Patients will be followed-up for 30 days post-surgery. Data will be entered into REDCap and a de-identified dataset will be help indefinitely by the PI. Plasma and urine samples will be kept in storage at -80ºC until time of assay.
